# microRNA-1827 represses MDM2 to positively regulate tumor suppressor p53 and suppress tumorigenesis

**DOI:** 10.18632/oncotarget.7088

**Published:** 2016-01-30

**Authors:** Cen Zhang, Juan Liu, Chunwen Tan, Xuetian Yue, Yuhan Zhao, Jiaping Peng, Xiaolong Wang, Saurabh V. Laddha, Chang S. Chan, Shu Zheng, Wenwei Hu, Zhaohui Feng

**Affiliations:** ^1^ Department of Radiation Oncology, Rutgers Cancer Institute of New Jersey, Rutgers, State University of New Jersey, New Brunswick, NJ 08903, USA; ^2^ Key Laboratory of Cancer Prevention and Intervention, China National Ministry of Education, Cancer Institute, Zhejiang University School of Medicine, Hangzhou, Zhejiang 310009, China; ^3^ Center for Systems Biology, Rutgers Cancer Institute of New Jersey, Rutgers, State University of New Jersey, New Brunswick, NJ 08903, USA; ^4^ Department of Pharmacology, Rutgers, State University of New Jersey, Piscataway, NJ 08854, USA

**Keywords:** tumor suppressor, p53, MDM2, negative feedback loop, microRNA

## Abstract

The tumor suppressor p53 plays a central role in tumor prevention. The E3 ubiquitin ligase MDM2 is the most critical negative regulator of p53, which binds to p53 and degrades p53 through ubiquitation. MDM2 itself is a transcriptional target of p53, and therefore, MDM2 forms a negative feedback loop with p53 to tightly regulate p53 levels and function. microRNAs (miRNAs) play a key role in regulation of gene expression. miRNA dysregulation plays an important role in tumorigenesis. In this study, we found that miRNA miR-1827 is a novel miRNA that targets *MDM2* through binding to the 3′-UTR of *MDM2* mRNA. miR-1827 negatively regulates MDM2, which in turn increases p53 protein levels to increase transcriptional activity of p53 and enhance p53-mediated stress responses, including apoptosis and senescence. Overexpression of miR-1827 suppresses the growth of xenograft colorectal tumors, whereas the miR-1827 inhibitor promotes tumor growth in mice in a largely p53-dependent manner. miR-1827 is frequently down-regulated in human colorectal cancer. Decreased miR-1827 expression is associated with high MDM2 expression and poor prognosis in colorectal cancer. In summary, our results reveal that miR-1827 is a novel miRNA that regulates p53 through targeting *MDM2*, and highlight an important role and the underlying mechanism of miR-1827 in tumor suppression.

## INTRODUCTION

Tumor suppressor p53 and its signaling pathway play a key role in tumor prevention [1–3]. Loss of p53 is critical for tumor initiation and progression, which has been clearly demonstrated by *p53* knockout mouse models and Li-Fraumeni syndrome in humans with germline *p53* heterozygous mutation [4–6]. The *p53* gene is frequently mutated in human tumors [7]. As a transcription factor, in response to stress, p53 is activated and accumulates in cells, which in turn initiates various cellular stress responses, including apoptosis, cell cycle arrest and senescence, through transcriptional regulation of its target genes to prevent tumorigenesis [1–3]. p53 protein levels and activities are tightly regulated by different mechanisms in cells to ensure its proper function [1–3].

E3 ubiquitin ligase MDM2 is the most critical negative regulator for p53. MDM2 binds to p53 and ubiquitinates p53 for proteasomal degradation [8, 9]. p53 protein levels are maintained at low steady-state levels in cells under normal and non-stressed conditions mainly through MDM2-mediated ubiquitination and degradation. Meanwhile, MDM2 is transcriptionally regulated by p53 [10, 11]. Thus, MDM2 forms an autoregulatory negative feedback loop with p53 [12, 13]. The fine balance of MDM2-p53 negative feedback loop is critical for p53 to maintain the appropriate levels and activities under both non-stressed and stressed conditions to exert its function in tumor suppression [12, 13]. The disruption of this balance is frequently observed in cancer, which contributes to tumorigenesis. For instance, MDM2 protein is frequently overexpressed and/or amplified in different types of tumors, which in turn inhibits p53 function and leads to tumorigenesis [14, 15]. Recent studies have also shown that a single nucleotide polymorphism (SNP) in the *MDM2* promoter (SNP309), which slightly increases MDM2 levels (by ~ 2-fold), significantly impacts upon tumorigenesis through attenuating p53 function in both human beings and animal models [16, 17].

microRNAs (miRNAs) are endogenously expressed small non-coding RNAs that play a critical role in regulating gene expression [18, 19]. miRNAs bind to target mRNAs to inhibit their translation and/or target them for cleavage and degradation. miRNA-binding sites are generally located at the 3′-untranslated regions (3′-UTRs) of target mRNAs [18, 19]. Recent studies have shown that miRNA dysregulation plays an important role in tumorigenesis by regulating many different biological processes, including cell proliferation, cell death, metastasis and metabolism [19, 20]. Growing evidence has shown that miRNAs interact closely with the p53 pathway. p53 regulates the expression of a number of miRNAs, which in turn mediates p53 function in tumor suppression [21–23]. On the other hand, p53 levels and function can be regulated by miRNAs. For instance, miR-125b, miR-504 and miR-30 can target p53 and down-regulate p53 protein levels and function [24–26]. miR-25, miR-32, miR-661 and miR-339–5p target MDM2 to up-regulate p53 protein levels and function [27–30]. Dysregulation of these miRNAs has been revealed as an additional important mechanism that leads to the impaired p53 function in cancer cells, which contributes to tumorigenesis [21–23].

In this study, we identified miR-1827 as a novel regulator for p53 by targeting *MDM2*. miR-1827 was recently reported to target *L-MYC*, and the nucleotide polymorphism for the miR-1827 binding site at the 3′-UTR of *L-MYC* is associated with increased risk for lung cancer, suggesting a potential role of miR-1827 in suppressing lung cancer [31]. The level of circulating miR-1827 in the serum was found to be decreased in ulcerative colitis patients who have an increased risk for colorectal cancer [32]. While these studies suggest a potential role of miR-1827 in cancer, the role and mechanism of miR-1827 in tumorigenesis is poorly defined. In this study, we found that miR-1827 binds to the 3′-UTR of *MDM2* to down-regulate MDM2 levels, which in turn enhances p53 levels and function. miR-1827 is frequently down-regulated and its expression is negatively associated with MDM2 expression in human colorectal cancer. Furthermore, decreased miR-1827 expression is associated with poor prognosis of colorectal cancer patients. These results reveal that miR-1827 is a novel miRNA that regulates the p53 function through targeting *MDM2*, and furthermore, suggest an important role and mechanism of miR-1827 in suppression of colorectal cancer.

## RESULTS

### miR-1827 down-regulates MDM2 to activate p53

**Figure 1:**
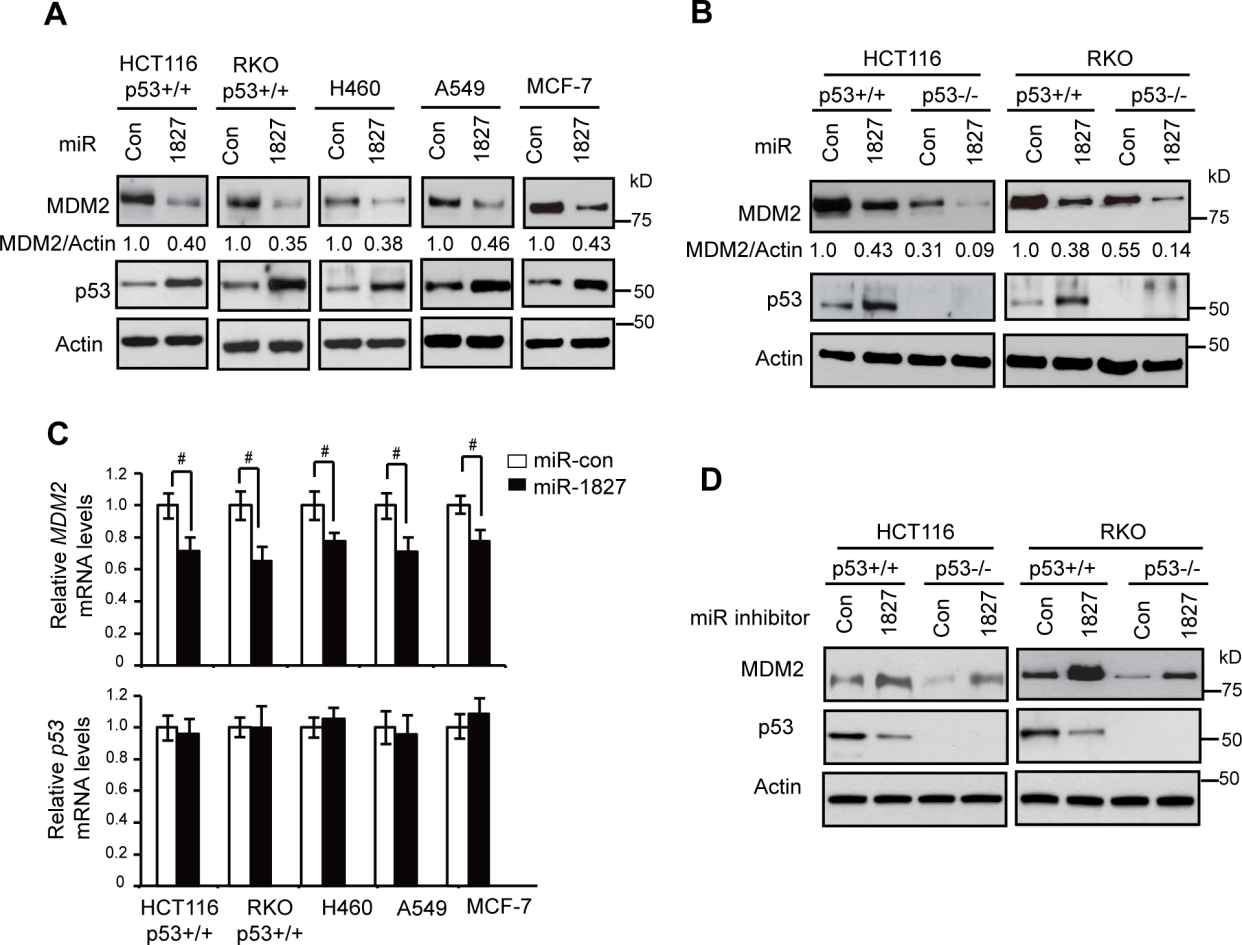
miR-1827 down-regulates MDM2 to activate p53 (**A**) miR-1827 down-regulated MDM2 protein levels and increased p53 protein levels in different human cells which express WT p53. Cells were transfected with miR-1827 mimic or scrambled miRNA control (miR-con). The MDM2 and p53 protein levels were measured at 24 h after transfection by western-blot assays. (**B**) miR-1827 negatively regulated MDM2 protein levels independently of p53 in HCT116 and RKO cells. HCT116 p53+/+, HCT116 p53–/–, RKO p53+/+ and RKO p53−/− cells were transfected with miR-1827 mimic or miR-con for western-blot assays. (**C**) miR-1827 negatively regulated the mRNA levels of *MDM2* but not *p53* in different cells. The mRNA levels of *MDM2* (upper panel) and *p53* (lower panel) were measured by Taqman real-time PCR in cells transfected with miR-1827 mimic or miR-con, and normalized with *Actin*. The mRNA levels of the *MDM2* and *p53* in control cells transfected with miR-con were designated as 1. Data are presented as mean ± SD (*n* = 3). ^#^*p* < 0.05; Student *t*-tests. (**D**) miR-1827 inhibitor increased MDM2 protein levels and reduced p53 protein levels in HCT116 and RKO cells. HCT116 p53+/+, HCT116 p53–/–, RKO p53+/+ and RKO p53−/− cells transfected with the miR-1827 inhibitor or scrambled control inhibitor were collected for western-blot assays at 24 h after transfection.

MDM2 is the most critical negative regulator for p53. To identify novel miRNAs that regulate MDM2 to affect p53 levels and function in human cells which could impact upon tumorigenesis, we performed a computational search for the potential miRNAs that target *MDM2*. Using two online miRNA target prediction tools, Targetscan (www.targetscan.org) and miRDB (mirdb.org), miR-1827 was predicted to be a potential miRNA that targets *MDM2* since 3′-UTR of *MDM2* contains multiple putative binding sites for miR-1827. To investigate whether miR-1827 can regulate MDM2, different human cell lines, including human colorectal HCT116 p53+/+ and RKO p53+/+ cells, human lung H460 and A549 cells, and human breast MCF-7 cells were transfected with miR-1827 mimic or scrambled miRNA control (miR-con). These cell lines all express wild-type (WT) p53. As shown in Figure 1A, miR-1827 mimic clearly reduced MDM2 protein levels in all of these cell lines. Furthermore, the down-regulation of MDM2 by miR-1827 in turn increased p53 protein levels in these cells. In addition to the above-mentioned p53 WT cell lines, the repression of MDM2 by miR-1827 was also observed in HCT116 p53−/− and RKO p53−/− cells, the isogenic p53-deficient cell lines for HCT116 p53+/+ and RKO p53+/+ cells, respectively (Figure 1B), which suggests that the down-regulation of MDM2 by miR-1827 is p53-independent. In addition to reducing MDM2 protein levels, the miR-1827 mimic also reduced *MDM2* mRNA levels in these above-mentioned cell lines, although the effect appears to be weaker compared with its effect on MDM2 protein levels (Figure 1A and 1C). In contrast, miR-1827 mimic did not affect the mRNA levels of *p53* (Figure 1C).

To test whether endogenous miR-1827 regulates MDM2, HCT116 p53+/+ and RKO p53+/+ cells were transfected with the miR-1827 inhibitor, single-stranded RNA oligonucleotides that match with mature miR-1827 sequences. As shown in Figure 1D, miR-1827 inhibitor led to the increased MDM2 protein levels and reduced p53 protein levels in both HCT116 p53+/+ and RKO p53+/+ cells. miR-1827 inhibitor also clearly increased MDM2 protein levels in HCT116 p53−/− and RKO p53−/− cells, suggesting that the induction of MDM2 by miR-1827 inhibitor is p53-independent. Collectively, these results demonstrate that miR-1827 is a bona fide miRNA targeting *MDM2*.

### miR-1827 binds to the 3′-UTR of *MDM2* to repress *MDM2*

**Figure 2:**
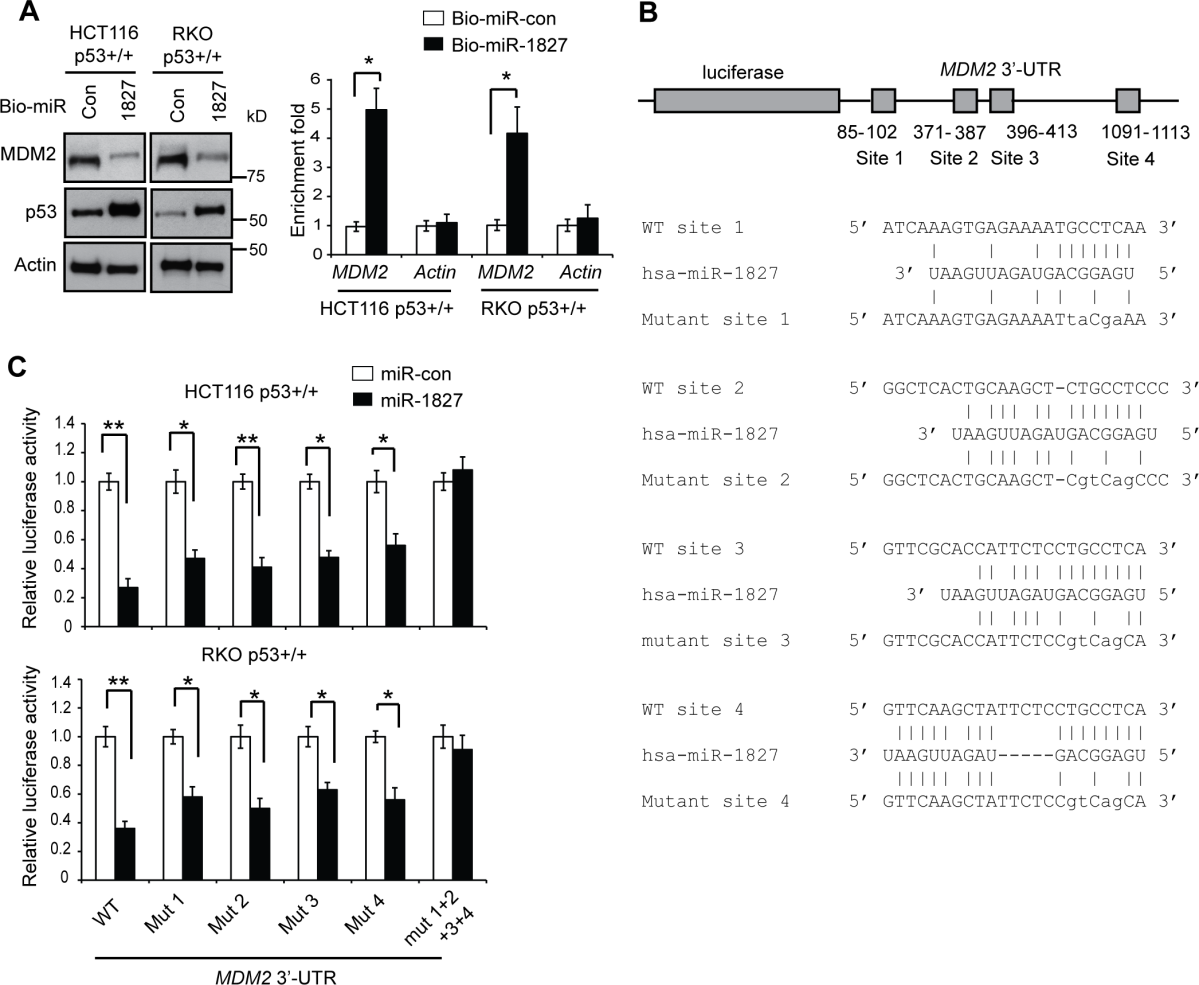
miR-1827 regulates MDM2 through binding to the 3′-UTR of *MDM2* (**A**) miR-1827 bound to *MDM2* mRNA in cells detected by miRNA pull-down assays. HCT116 p53+/+ and RKO p53+/+ cells were transfected with biotinylated miR-1827 (bio-miR-1827) or biotinylated miR-control (bio-miR-con), and collected at 24 h after transfection for miRNA pull-down assays. The levels of *MDM2* and *Actin* mRNAs bound to bio-miR-1827 or bio-miR-con were analyzed by Taqman real-time PCR assays. The mRNA levels were normalized to input (cellular RNA without incubation with beads) and then to *GAPDH*. Left panels: The down-regulation of MDM2 and up-regulation of p53 by bio-miR-1827 in cells detected by western-blot assays. Right panel: the enrichment fold with bio-miR-1827 relative to bio-miR-con for *MDM2* and *Actin* mRNA. (**B**) The schematic of the luciferase reporter containing the putative binding sites for miR-1827 at the 3′-UTR of *MDM2*. The sequences of miR-1827, its putative binding sites and their mutants at the 3′-UTR of *MDM2* were presented. The positions of putative binding sites are labeled. The drawing is not to scale. (**C**) miR-1827 negatively regulated *MDM2* through binding to the four binding sites at the 3′-UTR of *MDM2* as analyzed by luciferase reporter assays. HCT116 p53+/+ and RKO p53+/+ cells were transfected with luciferase reporter vectors containing WT or different mutant human *MDM2* 3′-UTR together with miR-1827 mimic or miR-con. Luciferase activities were measured at 24 h after transfection. In (A) and (C), data are presented as mean ± SD (*n* = 3). **p < 0.001; *p < 0.01; Student *t*-tests.

To obtain direct evidence for the interaction of miR-1827 with *MDM2* mRNA, miRNA pull-down assays were performed. HCT116 p53+/+ and RKO p53+/+ cells were transfected with biotinylated miR-1827 mimic or miRNA control. Biotinylated miR-1827 mimic was pulled down together with its associated mRNAs by using Streptavidin beads. The levels of MDM2 and Actin mRNAs bound to biotinylated miR-1827 mimic or miRNA control were analyzed by Taqman real-time PCR assays. As shown in Figure 2A, *MDM2* mRNA was significantly enriched in the miR-1827 pull-down compared with the miRNA control pull-down in both HCT116 p53+/+ and RKO p53+/+ cells. As a control, *Actin* mRNA was not enriched in the miR-1827 pull-down. These data strongly suggest that miR-1827 directly binds to *MDM2* mRNA *in vivo*.

Computational analysis using Targetscan and miRDB predicted that there are four putative binding sites for miR-1827 at the 3′-UTR of the human *MDM2* mRNA, which are clustered in the first 1.2 kb of *MDM2* 3′-UTR (Figure 2B). To investigate whether miR-1827 binds to these four putative binding sites to repress MDM2, luciferase reporter assays were employed. The first 1.6 kb of the *MDM2* 3′-UTR cDNA sequence containing these four sites were cloned and fused to the 3′ end of the luciferase reporter gene in a luciferase reporter vector. The reporter vector was transfected into HCT116 p53+/+ and RKO p53+/+ cells together with miR-1827 mimic. Compared with miR-control, miR-1827 mimic significantly decreased the luciferase activities of the reporter vectors in both cell lines (Figure 2C). While mutating any one of the four putative binding sites individually only partially repressed the inhibitory effect of miR-1827 on the luciferase activities, mutating these four binding sites simultaneously almost completely abolished the inhibitory effect of miR-1827 on the luciferase activities (Figure 2C), indicating that all these four predicated binding sites are functional binding sites for miR-1827. These results strongly suggest that miR-1827 targets *MDM2* through direct binding to the four binding sites in *MDM2* 3′-UTR.

### miR-1827 expression is frequently down-regulated in colorectal cancer and correlated with poor prognosis

**Figure 3:**
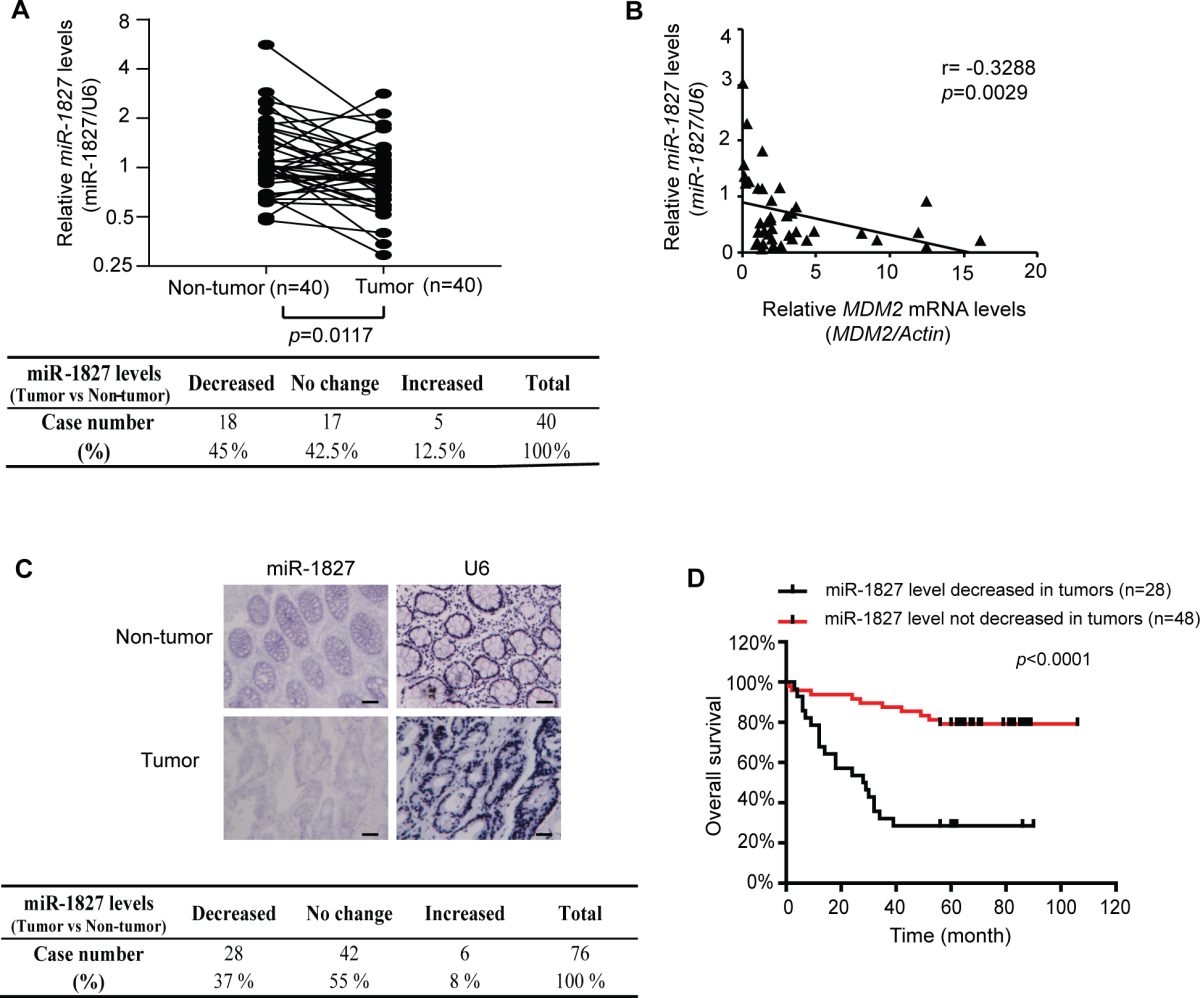
miR-1827 expression is frequently decreased, inversely correlated with *MDM2* expression in human colorectal cancer and associated with the poor prognosis of cancer patients (**A**) The expression of miR-1827 is frequently down-regulated in a cohort of colorectal cancer tissues compared with their paired adjacent non-tumor tissues measured by Taqman real-time PCR analysis (*n* = 18 out of 40; the cut-off is 2-fold change). The expression of *miR-1827* was normalized to the *U6* snRNA. *p* = 0.0117; paired Student *t*-test. Lower panel: summary of miR-1827 expression results in colorectal cancer specimens (*n* = 40). (**B**) A significant inverse correlation between the miR-1827 and *MDM2* expression levels in the cohort of colorectal cancer tissues (*n* = 40). The levels of *MDM2* mRNA were measured by Taqman real-time PCR assays and normalized with *Actin*. *p* = 0.0029; two-tailed correlation test. (**C**) The expression of miR-1827 is frequently down-regulated in the second cohort of colorectal cancer tissues compared with their paired adjacent non-tumor tissues analyzed by ISH (*n* = 28 out of 76). The *U6* snRNA was detected by ISH as an internal control. Upper panels: representative ISH staining images showing decreased expression of miR-1827 in a colorectal cancer tissue compared with its adjacent non-tumor tissue. Lower panel: summary of ISH staining results in colorectal cancer specimens (*n* = 76). *p* < 0.0001; χ ^2^ tests. Scale bars: 100 μm. (**D**) The decreased miR-1827 expression is significantly associated with the poor cancer-free survival in the second cohort of colorectal cancer patients. *p* < 0.0001; tumors showing decreased miR-1827 expression *vs.* tumors showing no decreased miR-1827 expression; log-rank (Mantel-Cox) test.

We further analyzed the expression of miR-1827 in specimens of human colorectal cancer. The levels of miR-1827 mRNA were analyzed by Taqman real-time PCR assays in a cohort of 40 pairs of de-identified human colorectal tumor specimens and their matched non-tumor adjacent tissues collected from the Rutgers Cancer Institute of New Jersey with no clinical outcome information (*n* = 40). As shown in Figure 3A, miR-1827 mRNA expression was frequently down-regulated in colorectal tumor samples; compared with the matched non-tumor adjacent colorectal tissues, 18 out of 40 tumor samples showed decreased miR-1827 expression (45%), whereas 17 tumor samples showed no clear change of miR-1827 expression and 5 tumor samples showed increased miR-1827 expression (*n* = 40; the cut-off is > 2 fold change; *p* = 0.0117). Furthermore, a significant inverse correlation between miR-1827 and *MDM2* mRNA expression in these colorectal cancer samples was observed (*p* = 0.0029) (Figure 3B), which strongly suggests that the down-regulation of miR-1827 increases MDM2 expression in human colorectal cancer. The expression of miR-1827 was further analyzed by *in situ* hybridization (ISH) staining in a second cohort of human colorectal tumors collected from Zhejiang University which has clinical outcome information (*n* = 76). Consistent with the results from the first cohort, miR-1827 mRNA expression was frequently down-regulated in the second cohort of colorectal tumor samples; compared with the matched non-tumor adjacent colorectal tissues, 28 out of 76 tumor samples showed decreased miR-1827 expression (37%), whereas 42 tumor samples showed no clear change of miR-1827 expression and 6 tumor samples showed increased miR-1827 expression (*n* = 76) (Figure 3C). Notably, decreased expression of miR-1827 is significantly correlated with poor cancer-free survival of patients in this cohort of colorectal cancer (*p* < 0.0001; Figure 3D).

### miR-1827 increases the transcriptional activity of p53

**Figure 4:**
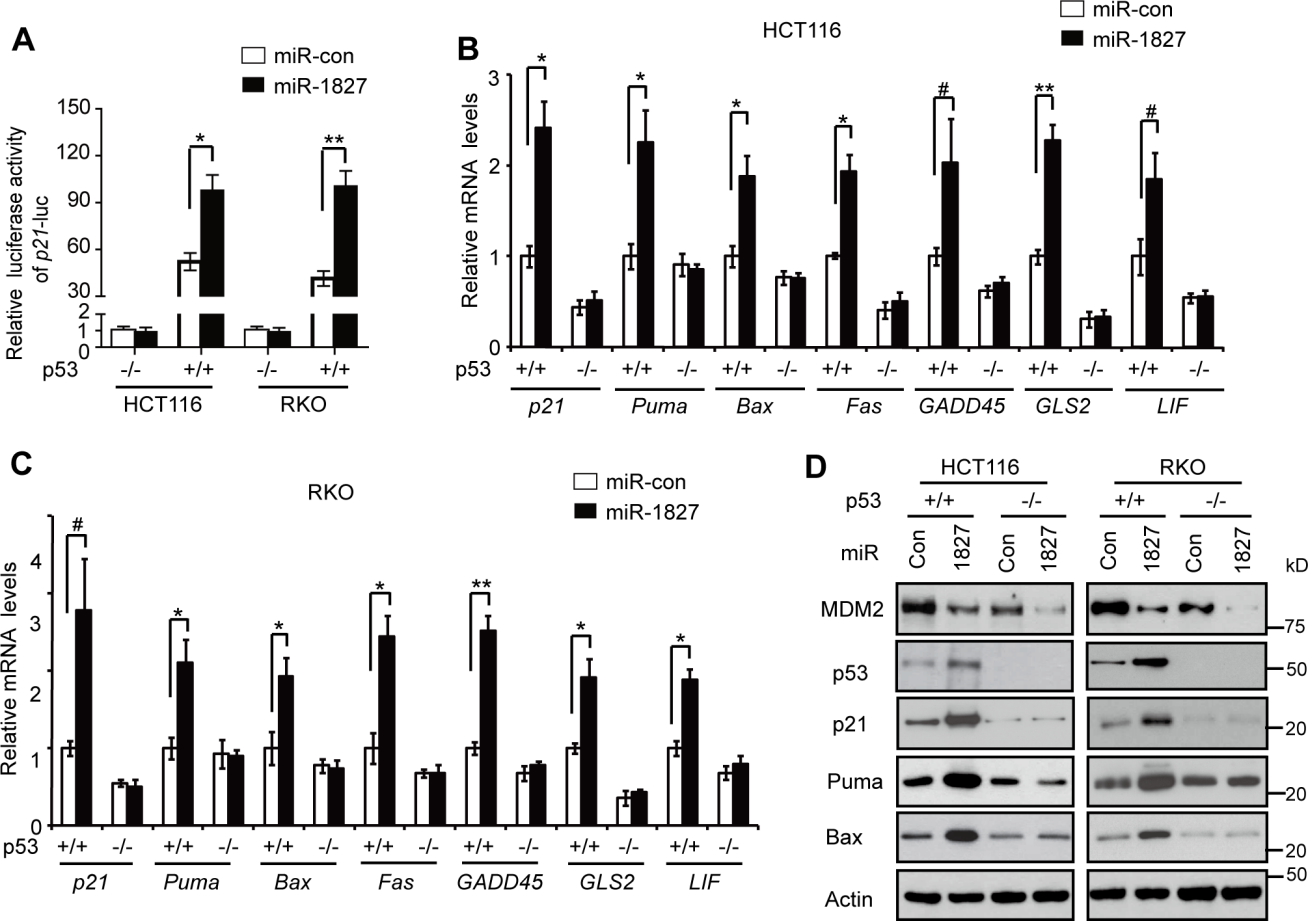
miR-1827 enhances the p53 transcriptional activity (**A**) miR-1827 induced the luciferase activity of the *p21*-Luc reporter vector in cells in a p53-dependent manner. p53+/+ and p53−/− HCT116 and RKO cells were transfected with miR-1827 mimic or miR-con together with the *p21*-Luc reporter vector which contains the p53 responsive element in the *p21* promoter for luciferase activity assays. (**B, C**) miR-1827 increased the mRNA levels of p53 target genes, including *p21*, *Puma*, *Bax*, *Fas*, *GADD45*, *GLS2* and *LIF*, in a p53-dependent manner in HCT116 (B) and RKO cells (C). (**D**) miR-1827 increased the protein levels of p21, Puma and Bax in a p53-dependent manner in HCT116 and RKO cells. In (B-D): p53+/+ and p53−/− HCT116 and RKO cells were transfected with miR-1827 mimic or miR-con, and the mRNA and protein levels of p53 target genes were analyzed at 24 h after transfection by Taqman real-time PCR (B, C) and western-blot assays (D), respectively. The mRNA levels of all genes were normalized to *Actin*. The mRNA levels of genes in p53+/+ cells transfected with miR-con were designated as 1. Data are presented as mean ± SD (*n* = 3). ^#^ *p* < 0.05; *p < 0.01; **p < 0.001; Student *t*-test.

As a transcription factor, p53 mainly exerts its function through directly binding to the p53 responsive elements (RE) in its target genes to transcriptionally regulate their expression [1–3]. Here, we investigated whether miR-1827 regulates the p53 transcriptional activity. To this end, the *p21* luciferase (*p21*-Luc) reporter vector which contains the p53 RE in the promoter of *p21* gene was used for luciferase reporter assays [28, 33]. As shown in Figure 4A, much higher luciferase activities of the *p21*-Luc reporter were observed in p53+/+ HCT116 and RKO cells compared with p53−/− HCT116 and RKO cells, respectively, confirming that the activation of *p21*-Luc reporter gene is dependent on p53 expression. Notably, compared with miR-control, miR-1827 mimic significantly induced the luciferase activities of *p21*-Luc in p53+/+ HCT116 and RKO cells but not in p53−/− HCT116 and RKO cells (Figure 4A), suggesting that miR-1827 activates p53 transcriptional activity.

We further investigated the effect of miR-1827 on the expression of a group of well-known p53 target genes, including *p21*, *Puma*, *Bax*, *Fas*, *GADD45*, *GLS2* and *LIF* [1–3, 34, 35]. Transfecting cells with miR-1827 mimic, which increased p53 protein levels, clearly induced the mRNA levels of *p21*, *Puma*, *Bax*, *Fas*, *GADD45*, *GLS2* and *LIF* in p53+/+ HCT116 and RKO cells but not in p53−/− HCT116 or RKO cells as detected by Taqman real-time PCR assays (Figure 4B and 4C). The p53-dependent induction of these genes by miR-1827 was confirmed at the protein level by western-blot analysis of selected genes, including *p21*, *Puma* and *Bax* (Figure 4D). These results together clearly show that miR-1827 enhances p53 transcriptional activity.

### miR-1827 enhances p53-mediated apoptosis in response to stress

**Figure 5:**
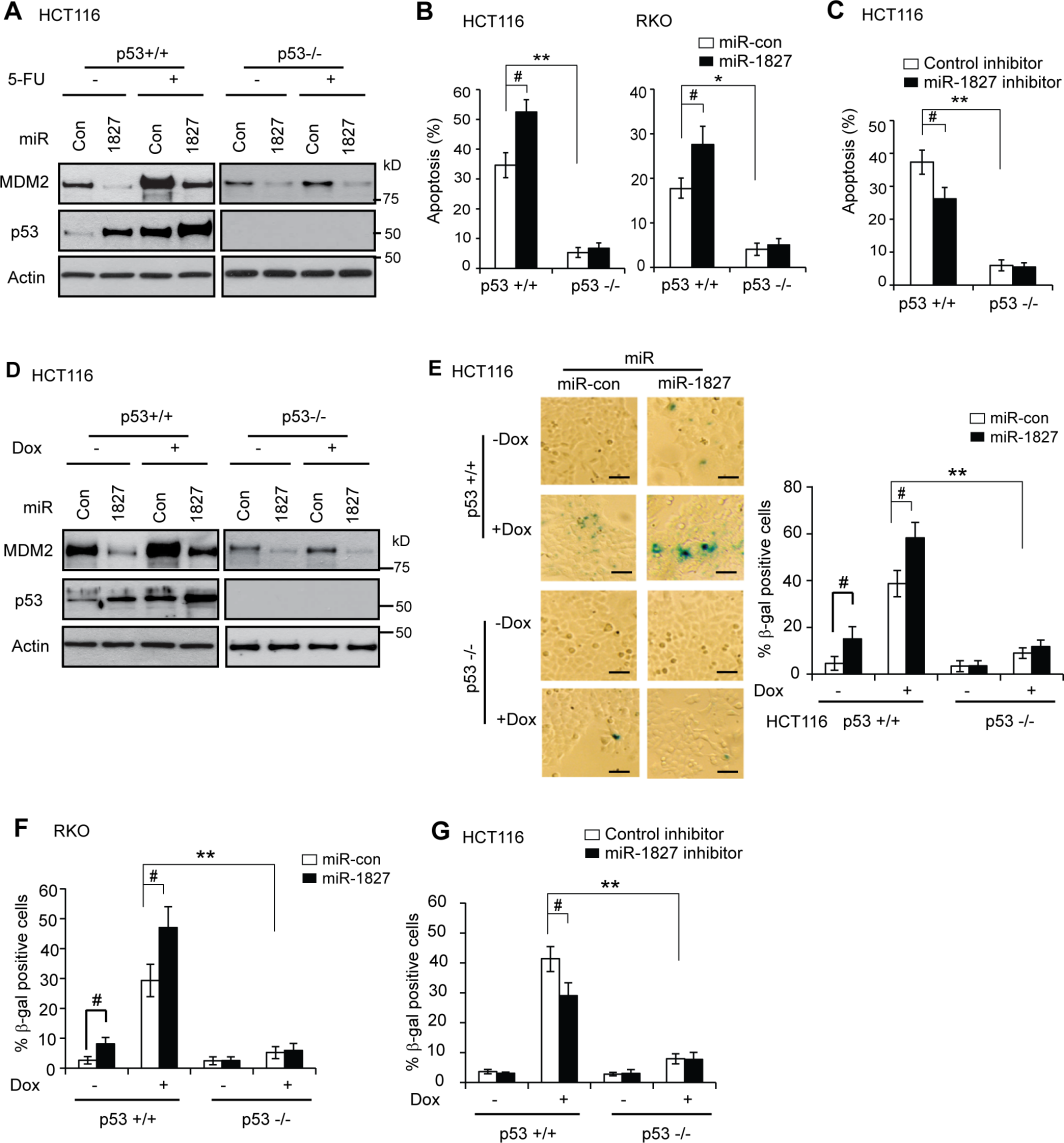
miR-1827 enhances p53-mediated apoptosis and senescence in response to stress (**A–C**) miR-1827 enhances p53-mediated apoptosis. (A) miR-1827 reduced MDM2 protein levels and increased p53 protein levels in HCT116 cells treated with 5-FU. p53+/+ and p53−/− HCT116 cells transfected with miR-1827 mimic or miR-con were treated with 5-FU for 12 h for western-blot assays. (B) miR-1827 enhanced p53-mediated apoptosis in HCT116 and RKO cells treated with 5-FU. (C) miR-1827 inhibitor reduced p53-mediated apoptosis in HCT116 cells treated with 5-FU. In B & C, cells were transfected with miR-1827 mimic or miR-con (B), or transfected with miR-1827 inhibitor or control inhibitor (C), and were then treated with 5-FU (300 μM for HCT116 cells and 500 μM for RKO cells, respectively). Apoptosis was measured by Annexin V staining in a flow cytometer at 36 h after treatment. Data are presented as mean ± SD (*n* = 4). ^#^*p* < *0.05*; *p < 0.01; **p < 0.001; Student *t*-test. (**D–G**) miR-1827 enhances p53-mediated senescence. (D) miR-1827 reduced MDM2 protein levels and increased p53 protein levels in HCT116 cells treated with Doxorubicin (Dox). p53+/+ and p53−/− HCT116 cells transfected with miR-1827 mimic or miR-con were treated with Doxorubicin for 12 h for western-blot assays. (E) miR-1827 enhanced p53-mediated senescence in HCT116 cells treated with Doxorubicin. (F) miR-1827 enhanced p53-mediated senescence in RKO cells treated with Doxorubicin. (G) miR-1827 inhibitor reduced p53-mediated senescence in HCT116 cells treated with Doxorubicin. In E–G, cells transfected with miR-1827 mimic (E, F) or miR-1827 inhibitor (G) were treated with Doxorubicin (100 nM for HCT116 cells and 300 nM for RKO cells, respectively) for 3 days before cellular senescence was detected by SA-b-gal staining. Left panels in E: represented images of SA-β-gal staining of senescent cells. Scale bar, 100 μm. E (Right panels) and F, G: the percentage of SA-β-gal positive cells. Data are presented as mean ± SD (*n* = 3). ^#^*p* < 0.05; **p < 0.001; Student *t*-test.

p53 can respond to various types of stress signals. In response to these stress signals, p53 is activated and p53 protein accumulate to a high level in cells, which in turn induces apoptosis and senescence as important mechanisms for tumor suppression [1–3]. It has been well-established that chemotherapeutic agent 5-Fluorouracil (5-FU), which is widely used for colorectal cancer treatment, can activate p53 and induce apoptosis in a largely p53-dependent manner in colorectal cancer cells [36, 37]. As shown in Figure 5A, in addition to regulating the basal MDM2 and p53 protein levels under non-stressed condition (without 5-FU treatment), miR-1827 mimic clearly decreased MDM2 protein levels and increased p53 protein levels in HCT116 p53+/+ cells treated with 5-FU. To investigate the impact of miR-1827 upon p53-mediated apoptosis induced by 5-FU, p53+/+ and p53−/− HCT116 cells transfected with miR-1827 mimic or miR-control were treated with 5-FU, and stained with annexin V-FITC and Propidium Iodide (PI) for flow cytometry analysis. As shown in Figure 5B, 5-FU treatment induced apoptosis in a highly p53-dependent manner in HCT116 cells; 5-FU induced significantly more apoptosis in HCT116 p53+/+ cells transfected with miR-control compared with HCT116 p53−/− cells transfected with miR-control. Notably, compared with miR-control, miR-1827 mimic significantly promoted 5-FU-induced apoptosis in HCT116 p53+/+ cells but not in HCT116 p53−/− cells (Figure 5B, left panel). Similar results were also observed in RKO cells (Figure 5B, right panel). These results demonstrated that miR-1827 enhances p53 function in mediating cellular apoptosis in response to stress.

To test whether endogenous miR-1827 regulates p53 function in mediating apoptosis in response to stress, p53+/+ and p53−/− HCT116 cells were transfected with miR-1827 inhibitor and then treated with 5-FU for apoptotic assays. Compared with control inhibitor, miR-1827 inhibitor significantly inhibited 5-FU-induced apoptosis (Figure 5C) in HCT116 p53+/+ but not in HCT116 p53−/− cells. This result suggests that inhibition of endogenous miR-1827 reduces the p53 function in mediating apoptosis in response to stress.

### miR-1827 enhances p53-mediated senescence in response to stress

Chemotherapeutic agent Doxorubicin can activate p53 and induce senescence in a largely p53-dependent manner in cells [38, 39]. To investigate whether miR-1827 affects the p53 function in inducing senescence, p53+/+ and p53−/− HCT116 cells transfected with miR-1827 mimic or miR-control were treated with Doxorubicin for three days, and senescent cells were detected by senescence associated β-galactosidase (SA-β-gal) staining. As shown in Figure 5D, miR-1827 clearly decreased MDM2 protein levels and increased p53 protein levels in cells treated with Doxorubicin. Doxorubicin induced senescence in a largely p53-dependent manner in HCT116 cells; significantly more senescent cells were observed in HCT116 p53+/+ cells treated with Doxorubicin compared with HCT116 p53−/− cells treated with Doxorubincin (Figure 5E). Furthermore, miR-1827 mimic significantly increased Doxorubicin-induced senescence in HCT116 p53+/+ cells but not in HCT116 p53−/− cells (Figure 5E). Similar results were also observed in RKO cells (Figure 5F). These results demonstrated that miR-1827 promotes the p53 function in mediating senescence in response to stress in cells.

To test whether endogenous miR-1827 regulates p53 function in mediating senescence in response to stress, p53+/+ and p53−/− HCT116 cells were transfected with the miR-1827 inhibitor and then treated with Doxorubicin for senescence assays. Compared with control inhibitor, miR-1827 inhibitor significantly inhibited Doxorubicin-induced senescence in HCT116 p53+/+ cells but not in HCT116 p53−/− cells (Figure 5G), suggesting that inhibition of endogenous miR-1827 reduces the p53 function in mediating senescence in response to stress.

### miR-1827 inhibits colorectal tumorigenesis *in vivo* in a largely p53-dependent manner

**Figure 6:**
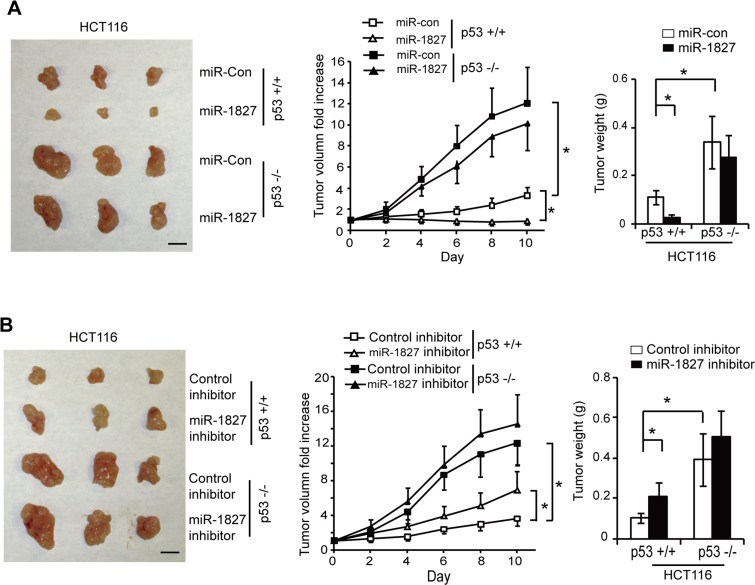
miR-1827 inhibits the growth of colorectal xenograft tumors *in vivo* in a largely p53-dependent manner (**A**) miR-1827 inhibited the growth of HCT116 xenograft tumors in nude mice in a largely p53-dependent manner. Xenograft tumors were established by s.c. injection of HCT116 p53+/+ and HCT116 p53−/− cells into nude mice. When tumor volumes reached ~60 mm^3^, tumors were injected with miR-1827 mimic or miR-con once every two days for 10 days. Left panel: representative tumors photographed at 10 days after treatment with miR-1827 mimic or miR-con. Scale bar: 10 mm. Middle panel: The growth curves of p53+/+ and p53−/− HCT116 tumors after miR-1827 injection. The relative volumes of the tumors before treatment at day 0 were designated as 1. Right panel: the weight of p53+/+ and p53−/− HCT116 tumors after treatment for 10 days. (**B**) miR-1827 inhibitor promoted the growth of HCT116 xenograft tumors in nude mice in a largely p53-dependent manner. p53+/+ and p53−/− HCT116 tumors (~60 mm^3^) were injected with miR-1827 inhibitor or control inhibitor once every two days for 10 days. Left panel: representative tumors photographed at 10 days after treatment. Scale bar: 10 mm. Middle panel: The growth curves of tumors after miR-1827 inhibitor injection. Right panel: the weight of tumors after treatment for 10 days. Data are presented as mean ± SD (*n* = 10 for each group). *p < 0.01.

Our results have shown that decreased expression of miR-1827 is associated with poor survival in colorectal cancer patients (Figure 3D). To investigate whether miR-1827 inhibits the colorectal tumorigenesis *in vivo* through p53 activation, p53+/+ and p53−/− HCT116 cells were injected (s.c.) into nude mice for xenograft tumorigenesis assays. When the tumor volume reached ~60 mm^3^, tumors were injected with miR-1827 mimic or miR-control once every 2 days for 10 days. As shown in Figure 6A, HCT116 p53−/− tumors injected with miR-control displayed a significant faster rate of growth compared with the HCT116 p53+/+ tumors injected with miR-control. This indicates that loss of p53 plays a critical role in promoting colorectal tumorigenesis, which is consistent with previous reports [25, 40–42]. Notably, compared with miR-control, miR-1827 mimic significantly inhibited the growth of HCT116 p53+/+ tumors (Figure 6A). Furthermore, much less pronounced inhibitory effect of miR-1827 on tumor growth was observed in HCT116 p53−/− tumors (Figure 6A). We further tested the effect of miR-1827 inhibitor on the growth of colorectal xenograft tumor. p53+/+ and p53−/− HCT116 xenograft tumors with ~60 mm^3^ volume were injected with control or miR-1827 inhibitor. Compared with the control inhibitor, miR-1827 inhibitor significantly promoted the growth of HCT116 p53+/+ xenograft tumor but showed a limited effect on HCT116 p53−/− xenograft tumors (Figure 6B). These results collectively demonstrate that miR-1827 inhibits the growth of colorectal tumors in a largely p53-dependent manner *in vivo*.

## DISCUSSION

MDM2 is the most critical negative regulator for p53, which has been clearly demonstrated by the embryonic lethality caused by MDM2 knockout in mice that can be rescued by p53 knockout [43, 44]. The fine balance between the MDM2 and p53 autoregulatory feedback loop is critical for p53 to exert its function in tumor suppression [12, 13]. Multiple mechanisms have been reported for MDM2 overexpression in tumors to impair p53 function. For example, *MDM2* DNA is found to be amplified in different types of cancers, including colorectal cancer [14, 15]. The SNP in the *MDM2* promoter (SNP309) results in the increased levels of MDM2 to promote tumorigenesis [16, 17]. The promoter demethylation, transcriptional activation and proteolytic degradation have also been reported to contribute to MDM2 overexpression in cancer [45–47]. Recently, several miRNAs, including miR-143/145, miR-192/194, miR-339–5p and miR-509-5p have been identified to regulate p53 levels and function through directly targeting *MDM2* [27, 29, 30, 48, 49]. miR-143/145 were reported to be down-regulated in head and neck squamous cell carcinoma [50]. miR-192/194 are down-regulated in multiple myeloma [48]. miR-339–5p is frequently down-regulated in colorectal cancer and breast cancer [29, 30]. Dysregulation of these miRNAs targeting *MDM2* has been suggested to be an additional mechanism that contributes to MDM2 overexpression in cancer cells. Thus, these miRNAs have become new and important regulators in the MDM2 and p53 negative feedback loop, adding a new layer of complex to p53 regulation in cells.

Our results in this study showed that miR-1827 is a novel miRNA that directly targets *MDM2* to regulate p53 protein levels and function, which in turn suppresses colorectal tumorigenesis. Through binding to the multiple sites at the 3′-UTR of *MDM2* mRNA, miR-1827 negatively regulates MDM2, which in turn induces p53 protein levels and activates p53 transcriptional activity and p53-mediated stress responses, including apoptosis and senescence. Overexpression of miR-1827 suppressed the growth of xenograft colorectal tumors, whereas inhibition of endogenous miR-1827 promoted the growth of xenograft colorectal tumors in mice in a largely p53-dependent manner. Our results showed that miR-1827 is frequently down-regulated in colorectal cancer, and its expression is negatively associated with MDM2 expression in colorectal cancer. Furthermore, the decreased expression of miR-1827 is associated with poor prognosis of colorectal cancer. Currently, the mechanism underlying the frequent down-regulation of miR-1827 in colorectal cancer cells is still unclear, which should be addressed by future studies. Interestingly, a recent study reported that the level of circulating miR-1827 in serum was down-regulated in ulcerative colitis patients, who have an increased risk of developing colorectal cancer, suggesting a potential role of miR-1827 in colorectal tumorigenesis [32]. Taken together, our results highlight a tumor suppressive function of miR-1827 in colorectal cancer through its up-regulation of p53 function. Considering the relative chemical simplicity of miRNA molecules, our results also suggest a potential application of miR-1827 in colorectal cancer therapy.

It is worth noting that miR-1827 also displayed a p53-independent inhibitory effect on tumor growth; miR-1827 mimic exhibited a certain level of inhibitory effect on HCT116 p53−/− tumors, although this effect was much less pronounced compared with its effect on HCT116 p53+/+ tumors. It has been known that a single miRNA can target several or many different genes [18, 19]. It was recently reported that *L-MYC* is a target of miR-1827, and miR-1827 may function as a tumor suppressor in lung cancer by targeting *L-MYC* [31]. In addition to MDM2 and p53, the regulation of *L-MYC* and other unidentified targets by miR-1827 could contribute to the p53-independent activities of miR-1827 in tumor suppression. miR-1827 was also found to be enriched in leukemia cell-derived exosomes [51]. These studies also suggest a potential role of miR-1827 in different types of cancer in addition to colorectal cancer through targeting different genes. Future studies to identify these additional targets and their possible crosstalk with the p53 signaling pathway would shed further light on the role and mechanism of miR-1827 in different cancer.

## MATERIALS AND METHODS

### Cells, transfection and treatment of cells

H460, A549, and MCF7 cells were obtained from American Type Culture Collection (ATCC). HCT116 p53+/+, HCT116 p53–/–, RKO p53+/+ and RKO p53−/− cells were generous gifts from Dr. Bert Vogelstein (John Hopkins University). miRNA mimic (Ambion, TX; 40 nM), miRNA inhibitor oligonucleotides (Ambion, TX; 100 nM) were transfected into cells using Lipofectamine 2000 (Invitrogen) as we previously described [25, 30].

### Luciferase reporter assays

The luciferase reporter vectors containing WT or mutant *MDM2* 3′-UTR were constructed as follows. The human *MDM2* 3′-UTR sequences (1.6 kp, 38–1617 nt from the start of 3′-UTR) containing four putative miR-1827 binding sites were amplified by PCR using following two primers: Forward primer 5′-ACT AGT TAT AAC CCT AGG AAT TTA GAC AAC C-3′ and reverse primer 5′-AAG CTT ACA TCA TTA CTC CCA TCC CTT AC-3′. The PCR products subcloned into the 3′ end of the firefly pMIR-luciferase reporter vector (Ambion) at HindIII and SpeI sites. The mutations at the putative miR-1827 binding sites were introduced using Quikchange II XL Site-Directed Mutagenesis Kit (Stratagene/Agilent Technologies). The firefly pGL2 *p21* luciferase reporter vector (*p21*-Luc), which contains the p53 RE in the *p21* promoter, was provided by Promega.

Luciferase reporter assays were performed as we previously described [25]. In brief, the firefly pMIR-luciferase reporter vectors or the pGL2 *p21* luciferase reporter vector (100 ng) were transfected into cells in 6-well plates together with miR-1827 mimic (40 nM) or miR-control as a negative control by using Lipofectamine 2000. pRL-SV40 vectors (5 ng) that express *Renilla* luciferase (Promega) were co-transfected to normalize the transfection efficiency. Luciferase activities were measured at 24 h after transfection by using the Dual Luciferase Assay kit (Promega). Firefly luciferase activities were normalized to *Renilla* luciferase activities.

### Western-blot assays

Standard Western blot assays were used to analyze protein expression as we previously described [52]. Following antibodies were used: anti-MDM2 (2A10; generous gift from Dr. Arnold Levine), anti-p53 (DO-1, Santa Cruz Biotechonology), anti-p21 (Ab-1, EMD Millipore), anti-Puma (Cell Signaling), anti-Bax (Santa Cruz Biotechonology), and anti-actin (Sigma). The band intensity on Western blots was quantified by digitalization of the X-ray film and analyzed with Image J software (NIH, Bethesda, MD, USA) and normalized to Actin.

### Tissue samples

The frozen human colorectal cancer samples and their matched adjacent non-tumor colorectal tissues were collected from Rutgers Cancer Institute of New Jersey with approved IRB (*n* = 40). The tissue microarrays (TMAs) are composed of 76 pairs of colorectal cancer samples and their matched adjacent non-tumor colorectal tissues collected from Cancer Institute of Zhejiang University with approved IRB. All these samples are de-identified. The first cohort does not have information of clinical outcome, and the second cohort has information of clinical outcome.

### Taqman real-time PCR analysis

The total RNA was purified by using a miRNeasy miRNA Isolation Kit (Qiagen) as we previously described [25]. The miR-1827 expression levels were determined by real-time PCR using Taqman primers and Taqman PCR master mixture (Applied Biosystems). The expression of miR-1827 was normalized with the expression of U6 snRNA. To detect the mRNA expression of *MDM2* and p53 target genes, cDNA was prepared with random primers using TaqMan reverse transcription kit (Applied Biosystems) as previously described [33, 53]. Gene expression levels were determined by real-time PCR using Taqman PCR master mixture and primers. The expression of genes was normalized to *Actin* gene.

### miRNA pull-down assays

Assays were performed as previously described [28]. Cells were transfected with biotinylated miR-1827 mimic or miR-control (40 nM; Integrated DNA Technologies) by using Lipofectamine 2000. At 24 h after transfection, cells were harvested in lysis buffer (20 mM Tris pH 7.5, 100 mM KCl, 5 mM MgCl2 and 0.3% NP-40). Cell lysates were then added to Streptavidin Dynabeads (Invitrogen) and incubated for 4 h at 4°C. The RNAs bound to the Streptavidin beads were extracted with Trizol (Invitrogen). The levels of *MDM2* and *Actin* mRNAs bound to biotinylated miR-1827 mimic or miR-control were analyzed by Taqman real-time PCR assays as described above. The mRNA levels were normalized to input (cellular RNA without incubation with beads) and then to *GAPDH* gene.

### miRNA *in situ* hybridization (ISH) analysis

ISH analysis was performed using double-DIG-labeled miRCURY locked nucleic acid (LNA) probes complementary to miR-1827 and U6 snRNA (Exiqon) as described [27, 54]. In brief, the tissue microarray (TMA) slides were hybridized with the DIG-labeled probes, and the DIG was detected with an anti-DIG antibody and an alkaline phosphatase-conjugated second antibody, using NBT-BCIP as the substrate. The ISH results were scored as previously described [55, 56]. In brief, signals in tumor cells were visually quantified using a scoring system from 0 to 9, multiplied intensity of signal, and percentage of positive cells (signal: 0 *=* no signal, 1 *=* weak signal, 2 *=* intermediate signal, and 3 *=* strong signal; percentage: 0 *=* 0%, 1 *=* < 25%, 2 *=* 25%–50%, and 3 *= >* 50%). Low, intermediate and high miR-1827 expression levels were defined as scores of 0–3, 3–6 and 6–9, respectively.

### Cellular apoptosis and senescence analysis

Cellular apoptosis assays were performed as we previously described [33, 57]. In brief, cells were treated with 5-FU (300 μM for HCT116 cells and 500 μM for RKO cells, respectively), and collected at 36 h after treatment. Cells were washed with PBS, stained with Alexa Fluor^®^ 488 annexin V/Dead Cell Apoptosis Kit (Life Technologies) before being analyzed in a flow cytometer (Beckman Coulter). For senescence assays, cells were treated with Doxorubicin (100 nM for HCT116 cells and 300 nM for RKO cells, respectively) for 3 days. Senescent cells were detected by senescence associated β-galactosidase assays using a Senescence β-Galactosidase Staining Kit (Cell Signaling) as we previously described [33].

### Xenograft tumorigenicity assays

Xenograft tumorigenicity assays were performed as previously described [57, 58]. In brief, p53+/+ and p53−/− HCT116 and RKO cells (5 *×* 10^6^ in 0.2 ml PBS) were injected (s.c.) into seven-week-old BALB/c nu/nu male athymic nude mice. When the volumes of xenograft tumors reach ~60 mm^3^, miR-1827 mimic or inhibitor (0.5 nM) was injected directly into the tumors every two days for 10 days (*n* = 10 per group). Tumors were injected with miR-control or control inhibitor for control groups. Tumor volume *=* ½ (length *×* width^2^). Tumor weight was measured after mice were sacrificed at the end of treatment of miR-1827 mimic or inhibitor. All mouse experiments were performed with the approval of the Institutional Animal Care and Use Committee of Rutgers University.

### Statistical analysis

Kaplan-Meier statistics were performed to analyze the significance of differences in survival of patients among different groups. The differences in tumor growth among groups were analyzed for statistical significance by analysis of variance, followed by Student's *t*-tests using GraphPad Prism software. All other *p* values were obtained using Student *t*-tests or χ^2^ tests. **p < 0.001; *p < 0.01; ^#^*p* < 0.05.
